# Prognostic value of uterine artery Doppler ultrasonography combined with serum PECAM-1 and PLGF levels and gut microbiota colony count for predicting outcomes in hypertensive disorder complicating pregnancy patients

**DOI:** 10.3389/fmed.2025.1745761

**Published:** 2026-01-12

**Authors:** Juan Zhang, Mingsheng Wu, Jing Chen, Yingbo Xu, Lin Yang

**Affiliations:** 1 Department of Nursing, Zhengzhou Health College, Zhengzhou, Henan, China; 2 Department of Obstetrics, Qingdao Municipal Hospital, Qingdao, Shandong, China; 3 Department of Clinical Medicine, Zhengzhou Health College, Zhengzhou, Henan, China

**Keywords:** gut microbiota, hypertensive disorder complicating pregnancy, PECAM-1, PlGF, prognostic value, uterine artery Doppler ultrasonography

## Abstract

This research aimed to determine the predictive value of a combined diagnosis of serum platelet endothelial cell adhesion molecule-1 (PECAM-1) and placental growth factor (PLGF) levels, gut microbiota count, and uterine artery Doppler ultrasonography in relation to pregnancy outcomes in patients with hypertensive disorders complicating pregnancy (HDCP). A total of 134 HDCP patients upon admission to our hospital from June 2022 to June 2024 were selected as the observation group, and 134 healthy prenatal checkups were selected as the control group during the same period. The uterine artery Doppler ultrasonography parameters, serum PECAM-1 and PLGF levels, and the number of gut microbiota colonies between both groups were compared. The results suggested that compared to the control group or good pregnancy outcome group, the observation group or adverse pregnancy outcome group had lower levels of serum PECAM-1 and PLGF, higher colony counts of *Escherichia coli* and Enterococcus, and lower colony counts of Lactobacillus and Bifidobacterium. The area under the curve for the combined diagnosis of adverse pregnancy outcomes in HDCP patients using uterine artery Doppler ultrasonography parameters, serum PECAM-1 and PLGF levels, counts of Lactobacillus, Enterococcus, Bifidobacterium, and *E. coli* was 0.878, which was upregulated compared to the individual diagnosis. Collectively, the combination of uterine artery Doppler ultrasonography, serum PECAM-1 and PLGF levels, and gut microbiota colony count has a high prognostic value for predicting adverse pregnancy outcomes in HDCP, which can provide clinical treatment options and is of great significance for improving pregnancy outcomes in pregnant women. This multimodal approach also supports nursing-led monitoring and patient education, facilitating early intervention in high-risk HDCP pregnancies.

## Introduction

Hypertensive disorder complicating pregnancy (HDCP) often presents clinical symptoms such as seizures and bleeding (1). The occurrence of HDCP is a major reason for maternal and neonatal morbidity and mortality (2). The physiological and pathological changes associated with HDCP depict major manifestations such as decline in cardiac output and systemic vascular spasm, which leads to reduced perfusion of multiple organs, resulting in ischemia and hypoxia. Consequently, this can cause a series of adverse outcomes such as slow development, chronic ischemia, and hypoxia in the fetus, and in severe cases, it may even lead to fetal stillbirth (3). Reports indicate that HDCP-triggered complications can affect pregnant women and their fetuses (4). Severe complications during pregnancy directly cause maternal and fetal mortality (5). Therefore, early diagnosis and proactive intervention of HDCP are of crucial clinical significance.

Due to the unique physiological and metabolic conditions of pregnant women, clinical diagnosis and treatment interventions for HDCP are very limited. Platelet endothelial cell adhesion molecule-1 (PECAM-1), a single-chain membrane surface protein, is associated with vascular endothelial cell infiltration (6). Related reports have demonstrated a correlation between PECAM-1 levels and HDCP (7, 8). Placental growth factor (PLGF) plays a role in facilitating vascular maturation and ameliorating hemodynamic function (9). The uterine artery is a primary source of maternal blood supply that meets the needs of fetal growth and development, directly impacting fetal intrauterine growth and perinatal prognosis (10). Imbalances in the human gut microbiota are closely related to the occurrence of cardiovascular diseases, particularly hypertension. During pregnancy, there will be corresponding periodic changes in the gut microbiota of mothers, such as an increase in the ratio of Firmicutes to Bacteroidetes, and marked differences in the abundance of various bacterial genera in the middle and late stages of pregnancy (11). Existing research has revealed that in the occurrence of HDCP, the overall microbiota structure and the dysbiosis of certain specific genera can, to some extent, reflect the health status of pregnant women in different conditions and may serve as useful clinical diagnostic and therapeutic biomarkers (12, 13). Nevertheless, the predictive value of these diagnostic methods for pregnancy outcomes in HDCP patients still needs further confirmation.

Therefore, this research aimed to clarify the predictive value of the combined diagnosis of serum PECAM-1 and PLGF levels, gut microbiota count, and uterine artery Doppler ultrasonography in relation to pregnancy outcomes in HDCP patients.

## Materials and methods

### Materials

A total of 134 HDCP patients upon admission to our hospital from June 2022 to June 2024 received selection as the observation group (OG), and 134 healthy individuals were selected as the control group (CG) during the same period. The OG had the following characteristics: Mean age of 31.05 (± 3.25) years; mean gestational age of 28.76 (± 2.54) weeks; and mean BMI of 23.46 (± 1.48) kg/m^2^. Inclusion criteria for the OG were as follows: (1) Meeting the diagnostic criteria related to HDCP, (2) singleton pregnancy, (3) presence of manifestations such as proteinuria and hypertension, and (4) no other pregnancy diseases, such as gestational diabetes mellitus. The CG had the following characteristics: Mean age of 30.83 (± 3.14) years; mean gestational age of 28.33 (± 2.47) weeks; mean BMI of 23.60 (± 1.53) kg/m^2^. Inclusion criteria for the CG were as follows: (1) Pregnant and lying-in women and their family members were aware of the research content and voluntarily signed informed consent and (2) all of them had completed prenatal checkups at our hospital. Exclusion criteria included the following: (1) presence of immune system disorders; (2) abnormal liver and kidney function; (3) presence of endometriosis, uterine fibroids, etc.; (4) twins or multiple births; (5) COVID-19 infection; and (6) abnormal blood pressure levels before pregnancy. This research received review and approval from the ethics committee of our hospital, and all pregnant and lying-in women and their family members signed informed consent.

### Measurement methods

1. Doppler ultrasound examination method. The Resona 7 color Doppler ultrasound diagnostic instrument (Mindray) is used in the study. The subjects were instructed to lie in a supine position. A probe frequency of 3.5–5.5 MHz was used, with a sound beam blood flow angle of < 600 and a sampling volume of 2 mm. Hand-held probes were utilized to scan the growth and development status of the fetus and to observe whether there were abnormalities in indicators such as fetal heart rate and umbilical artery blood flow. The instrument automatically calculated the umbilical artery pulsatility index (PI), resistance index (RI), and systolic-to-diastolic ratio (S/D) of blood flow velocity. Each parameter was continuously tested at least three times, and the average value was taken as the final result.
2. Serum PECAM-1 and PLGF detection methods. 5 mL of the elbow vein was drawn from all subjects on an empty stomach in the morning. The extracted blood was placed in a non-anticoagulant test tube and centrifuged in a low-temperature centrifuge for 10 min (3,500 r/min). The supernatant was collected and stored in a refrigerator (−80 °C). The concentration of PLGF content was detected through an AutoDELFIA 1,235 fully automatic time-resolved fluorescence immunoassay analyzer. The PECAM-1 level was detected using relevant reagent kits (Shanghai Jianglai Biotechnology Co., Ltd.) with an enzyme-linked immunosorbent assay.
3. Detection method for gut microbiota colony count. Fresh fecal specimens were collected from pregnant women in both groups, along with a sterile diluent, and inoculated on sterile culture medium. Specimen cultivation and colony counting were performed for *Escherichia coli*, Lactobacillus, Enterococcus, and Bifidobacterium. The results were expressed as logarithms of colony-forming units per gram of feces (lg CFU/g).
4. Classification criteria for HDCP severity. Severe preeclampsia group: Diastolic blood pressure of ≥110 mmHg, systolic blood pressure of ≥ 160 mmHg, and proteinuria of ≥2.0 g/24 h [random urine protein ≥ (+++)]. Mild preeclampsia group: Diastolic blood pressure of ≥90 mmHg, systolic blood pressure of ≥140 mmHg, and proteinuria of ≥0.3 mg/24 h (random urine protein tested positive). HDCP group: Diastolic blood pressure of ≥90 mmHg, systolic blood pressure of ≥140 mmHg, and proteinuria had negative values.

### Observation indicators

1. The uterine artery Doppler ultrasonography parameters, including pulsatility index (PI), resistive index (RI), and systolic-to-diastolic pressure (S/D) between both groups, were compared.
2. The serum PECAM-1 and PLGF levels between both groups were compared.
3. The number of gut microbiota colonies between both groups was compared.
4. The uterine artery Doppler ultrasonography parameters (RI, PI, S/D), serum PECAM-1, PLGF levels, and gut microbiota colony count in pregnant women with different types of HDCP were compared.
5. According to the occurrence of adverse events in pregnancy outcomes (such as premature birth, fetal distress, stillbirth, and spontaneous abortion), all patients were divided into an adverse pregnancy outcome group (78 cases) and a good pregnancy outcome group (56 cases). The uterine artery Doppler ultrasonography parameters (RI, PI, S/D), serum PECAM-1, PLGF levels, and gut microbiota colony count between both groups were compared.
6. The predictive value was analyzed by plotting receiver operating characteristic (ROC) curves.

### Statistical analysis

SPSS 27.0 and GraphPad Prism 9.0 was used to analyze data. Measurement data conforming to a normal distribution received representation by (x ± s), followed by an independent samples *t*-test for comparison between two groups. One-way analysis of variance was performed to evaluate intergroup comparisons, and the LSD-t test was used for pairwise comparisons. Counting data received expression as (%) and were subjected to a chi-squared test. ROC analysis was performed to assess the predictive value of uterine artery Doppler ultrasonography combined with serum PECAM-1, PLGF levels and gut microbiota colony count for adverse pregnancy outcomes in HDCP, and prognostic efficacy was evaluated using area under the curve (AUC) values along with its 95% confidence interval (CI). To account for potential confounding factors, multivariate logistic regression analysis was performed with adverse pregnancy outcome as the dependent variable, and age, parity, BMI, and the key indicators (uterine artery RI, PI, S/D, serum PECAM-1, PLGF, and gut microbiota counts) as independent variables. Due to their strong predictive power and to facilitate clinical interpretation, uterine artery Doppler parameters (RI, PI, and S/D) were dichotomized using established clinical cutoff values (RI > 0.80, PI > 1.60, S/D > 2.50) for inclusion in the multivariate logistic regression model. A *p*-value of < 0.05 indicated a statistically significant difference.

## Results

### There are no marked differences between the CG and OG in terms of general data

The comparison of general information between CG and OG demonstrated no statistical significance (*p* > 0.05; Table 1), indicating comparability.

**Table 1:** General data of HDCP patients in CG and OG.

Groups	*N*	Age (years)	Gestational weeks (weeks)	BMI (kg/m^2^)
CG	134	30.83 ± 3.14	28.33 ± 2.47	23.60 ± 1.53
OG	134	31.05 ± 3.25	28.76 ± 2.54	23.46 ± 1.48
*χ*^2^/t		0.537	0.314	0.946
*p*		0.592	0.754	0.345

### Comparison of uterine artery Doppler ultrasonography parameters between the CG and OG

**Figure 1:**
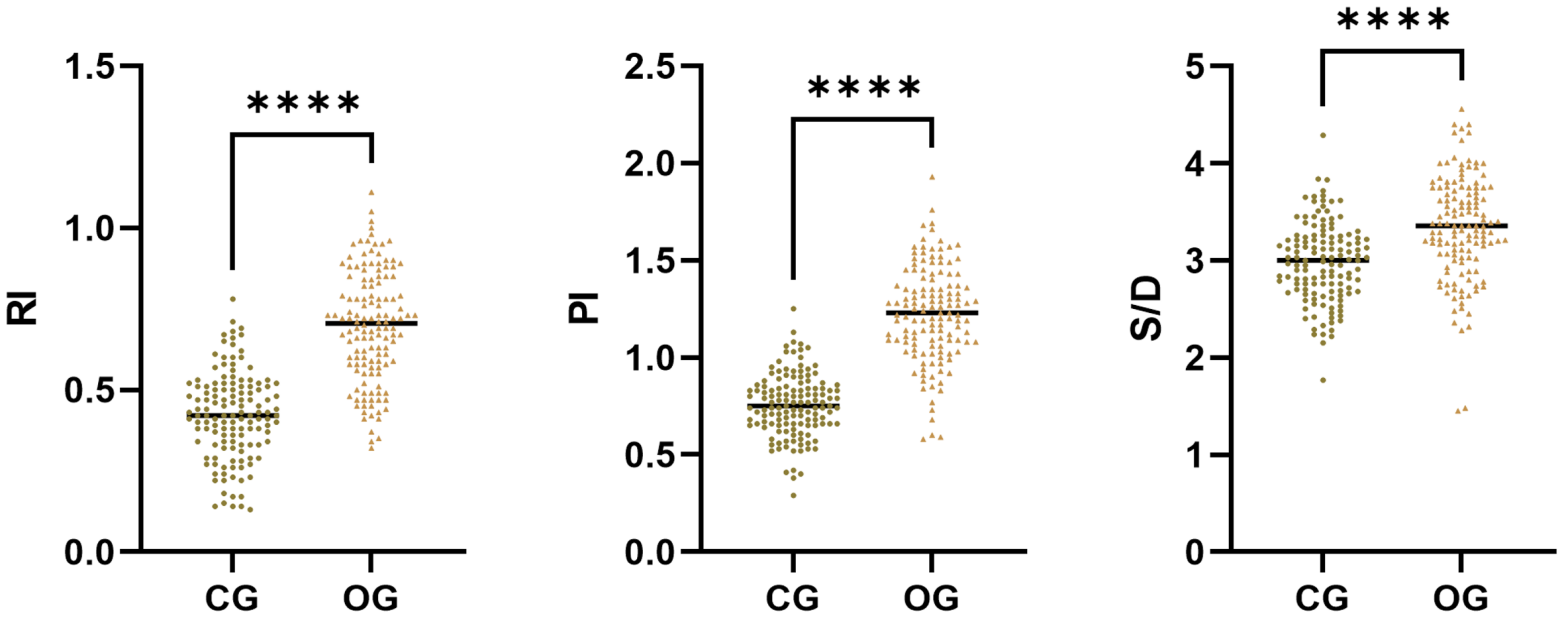
Uterine artery Doppler ultrasonography parameters in CG and OG. Versus CG, ^****^p < 0.0001.

The RI, PI, and S/D in OG illustrated upregulation in comparison to those in the CG (*p* < 0.05; Figure 1).

### Comparison of serum PECAM-1 and PLGF levels between CG and OG

**Figure 2:**
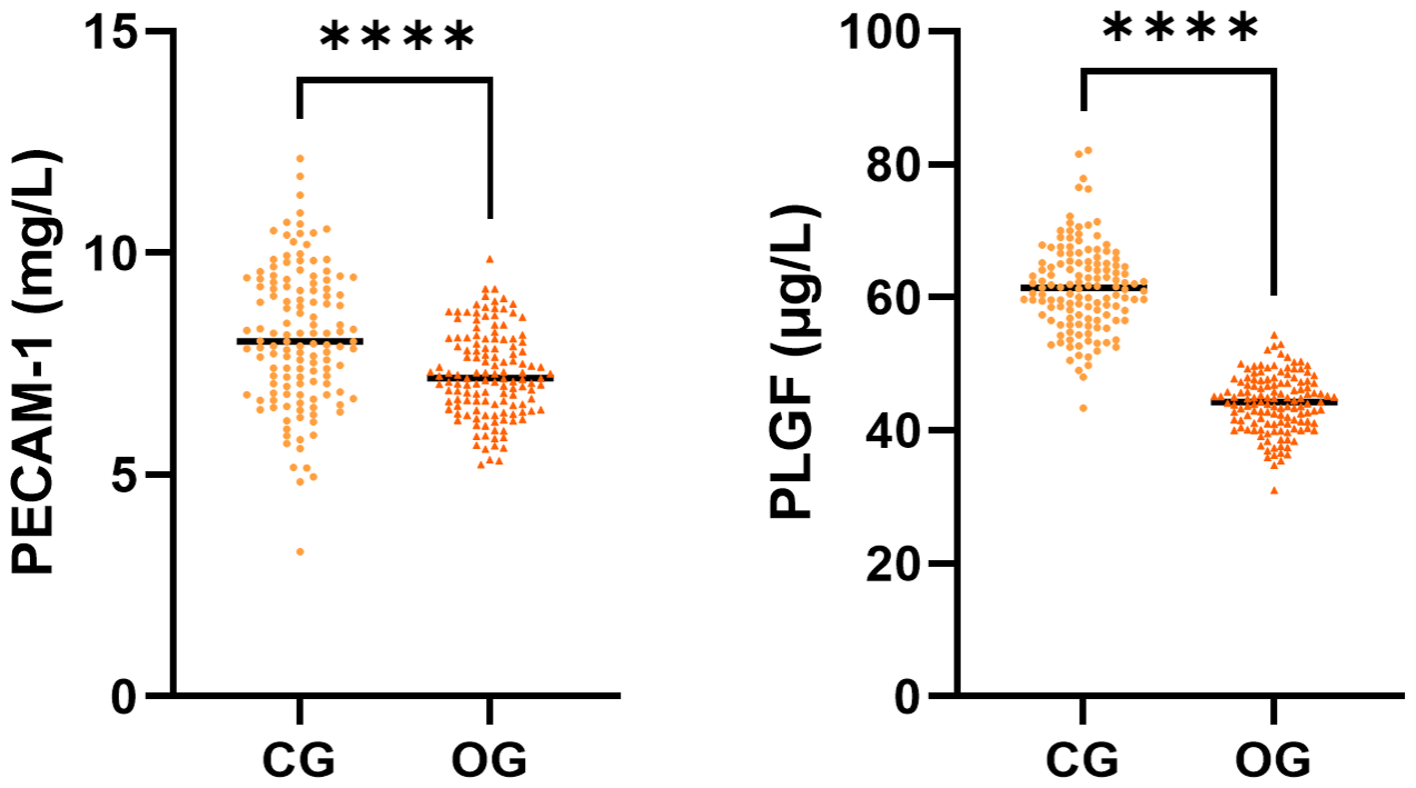
Serum PECAM-1 and PLGF levels in CG and OG. Versus CG, ^****^p < 0.0001.

The serum PECAM-1 and PLGF levels in the OG illustrated downregulation in comparison to those in CG (*p* < 0.05, Figure 2).

### Comparison of gut microbiota distribution between CG and OG

**Figure 3:**
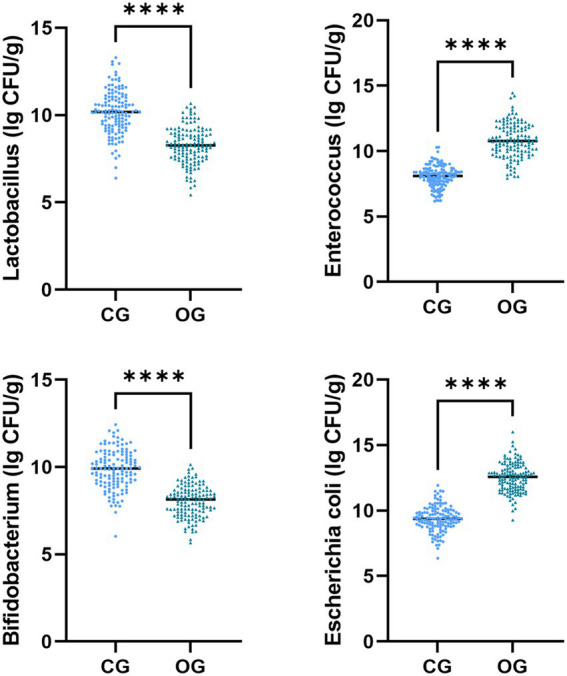
Gut microbiota distribution in CG and OG. Versus CG, ^****^p < 0.0001.

The *Escherichia coli* and Enterococcus colony counts in the OG illustrated upregulation in comparison to those in the CG (*p* < 0.05), while Lactobacillus and Bifidobacterium colony counts in the OG illustrated downregulation in comparison to those in the CG (*p* < 0.05, Figure 3).

### Comparison of uterine artery ultrasonography parameters, serum PECAM-1 and PLGF levels, and gut microbiota colony count in pregnant women with different types of HDCP

**Figure 4:**
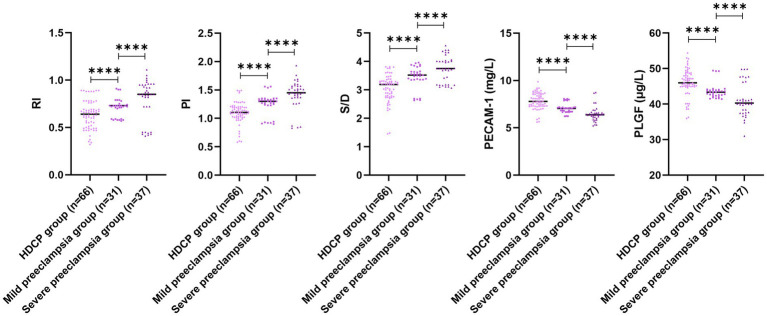
Uterine artery ultrasonography parameters and serum PECAM-1 and PLGF levels in pregnant women with different types of HDCP. Versus the mild preeclampsia group, ^****^p < 0.0001.

**Figure 5:**
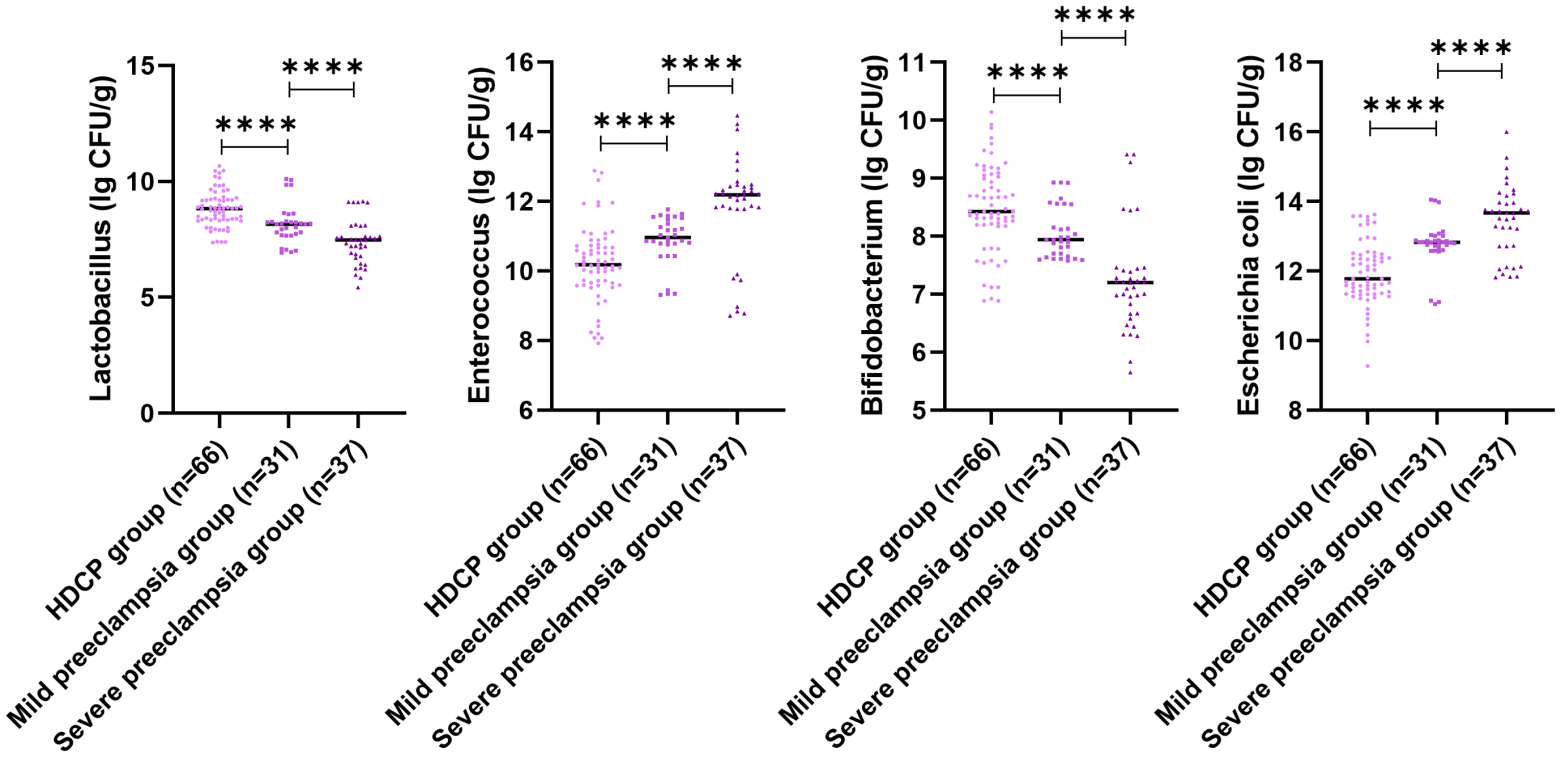
Gut microbiota colony count in pregnant women with different types of HDCP. Versus the mild preeclampsia group, ^****^p < 0.0001.

In comparison with the mild preeclampsia group, the HDCP group illustrated elevated serum PECAM-1 and PLGF levels, and depleted RI, PI, and S/D; in comparison with the mild preeclampsia group, the severe preeclampsia group illustrated depleted serum PECAM-1 and PLGF levels, and elevated RI, PI, and S/D (*p* < 0.05; Figure 4). In comparison with the mild preeclampsia group, the HDCP group illustrated depleted *E. coli* and Enterococcus colony counts, and elevated Lactobacillus and Bifidobacterium colony counts; in comparison with the mild preeclampsia group, the severe preeclampsia group illustrated elevated *E. coli* and Enterococcus colony counts and depleted Lactobacillus and Bifidobacterium colony counts (*p* < 0.05, Figure 5).

### Comparison of uterine artery ultrasonography parameters, serum PECAM-1 and PLGF levels, and gut microbiota colony count for different pregnancy outcomes

**Figure 6:**
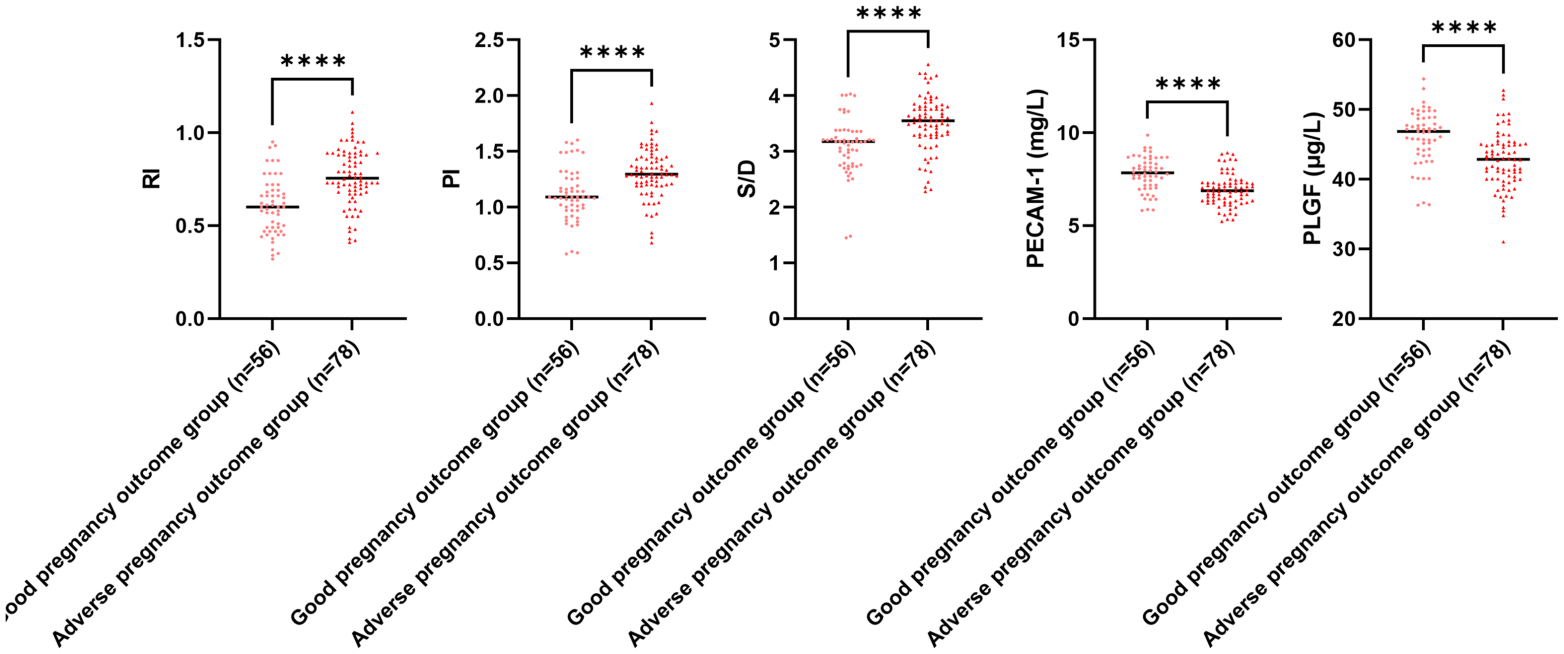
Uterine artery ultrasonography parameters and serum PECAM-1 and PLGF levels for different pregnancy outcomes. Versus the good pregnancy outcome group, ^****^p < 0.0001.

**Figure 7:**
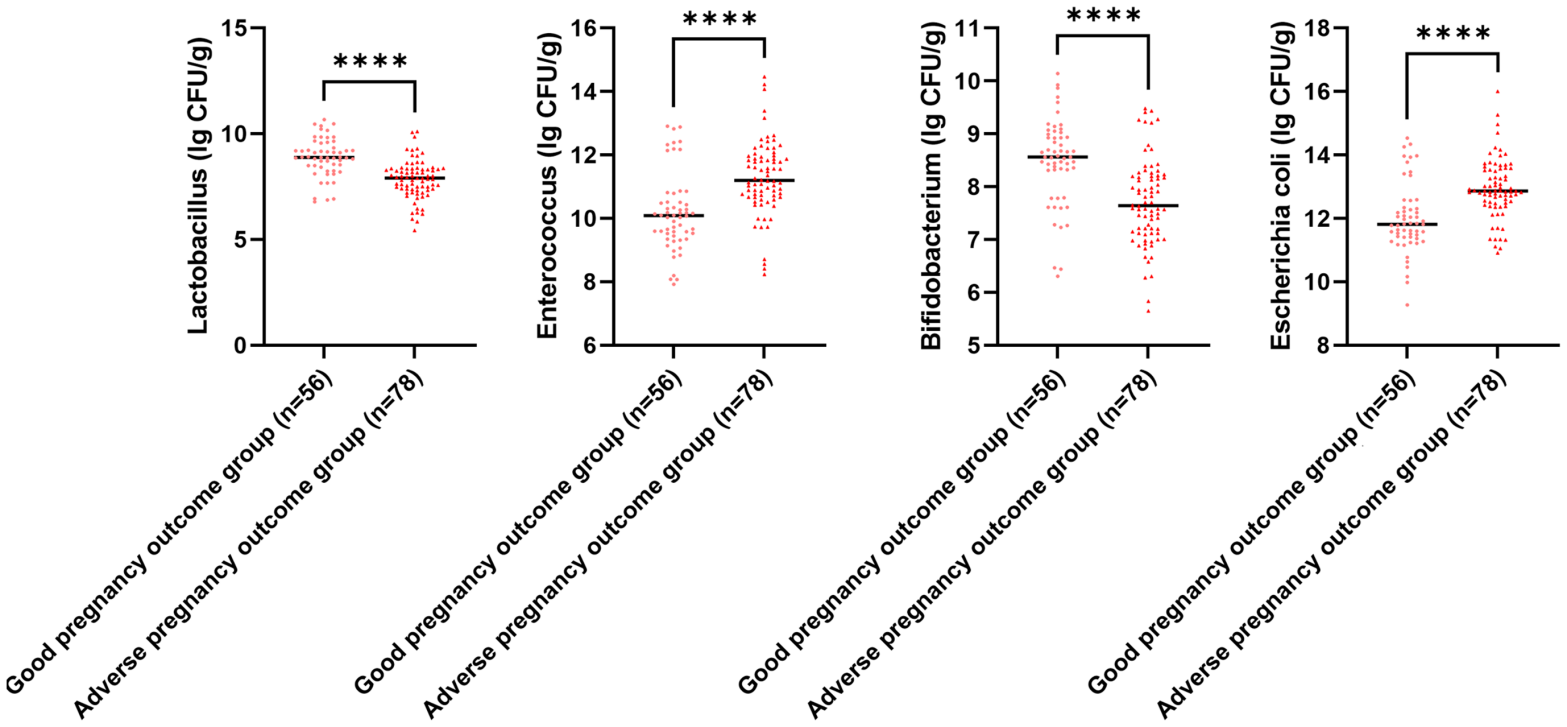
Gut microbiota colony count for different pregnancy outcomes. Versus the good pregnancy outcome group, ^****^p < 0.0001.

The serum PECAM-1 and PLGF levels in the adverse pregnancy outcome group illustrated downregulation in comparison to those in the good pregnancy outcome group, while RI, PI, and S/D in the adverse pregnancy outcome group illustrated upregulation in comparison to those in the good pregnancy outcome group (*p* < 0.05; Figure 6). The *E. coli* and Enterococcus colony counts in the adverse pregnancy outcome group illustrated upregulation in comparison to those in the good pregnancy outcome group, while Lactobacillus and Bifidobacterium colony counts in the adverse pregnancy outcome group illustrated downregulation in comparison to those in the good pregnancy outcome group (*p* < 0.05, Figure 7).

### ROC analysis on the prognostic efficacy of different diagnostic methods for pregnancy outcomes in pregnant women with HDCP

**Figure 8:**
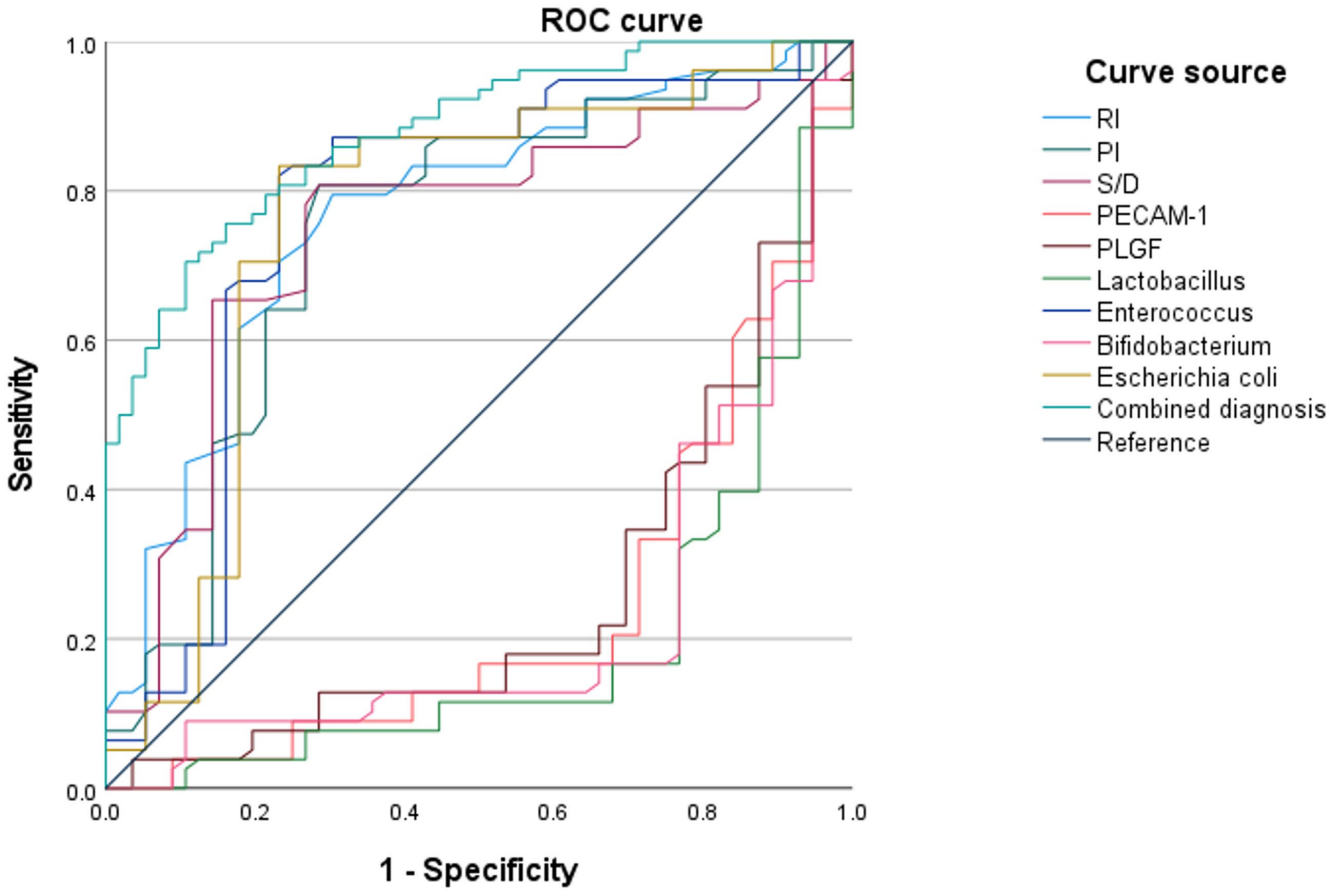
Prognostic efficacy of different diagnostic methods for pregnancy outcomes in pregnant women with HDCP.

The ROC curves were plotted using adverse pregnancy outcomes as positive samples and good pregnancy outcomes as negative samples. The results depicted that the AUC for the combined diagnosis of RI, PI, S/D, serum PECAM-1 and PLGF levels, Lactobacillus, Enterococcus, Bifidobacterium, and *E. coli* for predicting adverse pregnancy outcomes in HDCP was 0.878 (95% CI: 0.823–0.934), which illustrated upregulation in comparison to that of individual diagnosis (Figure 8).

### Multivariate logistic regression analysis of adverse pregnancy outcomes

To identify factors independently associated with adverse pregnancy outcomes while adjusting for potential confounders, a multivariate logistic regression analysis was performed. The model included maternal age, parity, BMI, and the key investigated parameters. Uterine artery Doppler indices were dichotomized based on clinically relevant cutoffs to optimize model stability and interpretation (RI > 0.80, PI > 1.60, S/D > 2.50). The results are presented in Table 2.

**Table 2:** Multivariate logistic regression analysis of factors associated with adverse pregnancy outcomes in HDCP patients.

Variable	B	SE	Wald *χ*^2^	*p* value	aOR	95% CI for aOR
Demographic and clinical factors						
Age (per 5-year increase)	0.18	0.13	1.92	0.166	1.20	(0.93–1.54)
Parity (Multiparous vs. Nulliparous)	0.35	0.29	1.46	0.227	1.42	(0.81–2.50)
BMI at enrollment (kg/m^2^)	0.05	0.10	0.25	0.617	1.05	(0.86–1.28)
Uterine artery doppler indices						
RI (>0.80 vs. ≤0.80) †	3.81	0.75	25.78	**<0.001**	45.15	(10.36–196.78)
PI (>1.60 vs. ≤1.60) †	2.05	0.62	10.93	**0.001**	7.77	(2.31–26.18)
S/D (>2.50 vs. ≤2.50)†	1.42	0.58	5.99	**0.014**	4.14	(1.33–12.91)
Serum biomarkers						
PECAM-1 (per 10 ng/mL decrease)	−0.08	0.02	16.00	**<0.001**	0.92	(0.89–0.96)
PLGF (per 100 pg./mL decrease)	−0.02	0.004	25.00	**<0.001**	0.98	(0.97–0.99)
Gut microbiota (lg CFU/g)						
*Lactobacillus* (per 1 unit decrease)	−0.85	0.25	11.56	**0.001**	0.43	(0.26–0.70)
*Enterococcus* (per 1 unit increase)	0.73	0.22	11.02	**0.001**	2.08	(1.35–3.20)
*Bifidobacterium* (per 1 unit decrease)	−0.29	0.19	2.33	0.127	0.75	(0.52–1.08)
*Escherichia coli* (per 1 unit increase)	0.31	0.18	2.96	0.085	1.36	(0.96–1.94)

The dependent variable was adverse pregnancy outcome (1 = yes, 0 = no).

†The cutoff values for uterine artery Doppler indices (RI > 0.80, PI > 1.60, S/D > 2.50) were chosen based on clinical relevance and commonly referenced thresholds in the obstetrics literature for predicting adverse outcomes. These variables were entered into the model as dichotomous (binary) variables.

Variables with a *p*-value of < 0.05 are considered statistically significant and are highlighted in bold. The analysis demonstrates that elevated uterine artery Doppler indices, lower serum PECAM-1 and PLGF levels, decreased Lactobacillus, and increased Enterococcus are independent predictors of adverse pregnancy outcomes in HDCP patients after adjusting for baseline maternal characteristics.

After adjustment, elevated uterine artery Doppler indices emerged as strong independent predictors. Specifically, an RI value of >0.80 was associated with a 45.15-fold increased risk [adjusted odds ratio (aOR) = 45.15, 95% CI: 10.36–196.78, *p* < 0.001]. Similarly, PI > 1.60 and S/D > 2.50 conferred significantly higher risks (aOR = 7.77, 95% CI: 2.31–26.18, *p* = 0.001; and aOR = 4.14, 95% CI: 1.33–12.91, *p* = 0.014, respectively).

Lower levels of serum biomarkers also retained independent significance. Each 10 ng/mL decrease in PECAM-1 was associated with an 8% increase in the odds of experiencing an adverse pregnancy outcome (aOR = 0.92, 95% CI: 0.89–0.96, *p* < 0.001). Similarly, each 100 pg./mL decrease in PLGF levels increased the odds by 2% (aOR = 0.98, 95% CI: 0.97–0.99, p < 0.001).

Regarding gut microbiota, after controlling for other variables, a decrease in Lactobacillus abundance (per 1 lg CFU/g) was independently associated with a 57% reduction in the odds of a favorable outcome (aOR = 0.43, 95% CI: 0.26–0.70, *p* = 0.001). Conversely, an increase in Enterococcus (per 1 lg CFU/g) was associated with more than a doubling of the risk (aOR = 2.08, 95% CI: 1.35–3.20, *p* = 0.001). The abundances of Bifidobacterium and *E. coli* were not retained as statistically significant independent predictors in the full model (*p* > 0.05).

## Discussion

In recent years, the incidence rate of HDCP disease has been on the rise year by year, which seriously threatens the life safety of pregnant women and fetuses. Thus, it is crucial to develop a timely and accurate examination method to diagnose conditions to ameliorate adverse pregnancy outcomes.

Doppler ultrasonography is an auxiliary examination method for the clinical detection of blood vessels. The supply of maternal blood to the uterus is a vital element in ensuring fetal safety. Embryos utilize the placenta to complete the supply of basic substances for growth, metabolism, and to eliminate metabolic products. Through examining uterine artery Doppler ultrasonography, the blood supply to the uterus and placenta can be evaluated, with the advantages of non-invasiveness, simplicity, strong objectivity, and good repeatability, and it can be widely accepted by patients (14). Herein, RI, PI, and S/D in the OG illustrated upregulation in comparison to those in CG, and RI, PI, and S/D in the adverse pregnancy outcome group illustrated upregulation in comparison to those in the good pregnancy outcome group. Research has depicted that a continuous increase in PI can reflect abnormal changes in vascular resistance; through monitoring dynamic changes in S/D values, the blood flow status of the uterus and placenta at a certain stage can receive a clear presentation; an increase in RI value is a preferred indicator for predicting severe intrauterine growth restriction in high-risk populations (15, 16). Herein, in comparison with the mild preeclampsia group, the HDCP group illustrated depleted RI, PI, and S/D, and the severe preeclampsia group illustrated elevated RI, PI, and S/D, indicating a certain relationship between abnormal parameters of uterine artery hemodynamics and the severity of HDCP patients. Furthermore, our multivariate analysis confirmed that an elevated RI (>0.80) was the strongest independent predictor, associated with a more than 45-fold increased risk of adverse outcome (aOR = 45.15, 95% CI: 10.36–196.78).

PECAM-1, usually distributed in cells such as platelets and endothelial cells, can directly reflect the functional status of the vascular endothelium (17). Herein, serum PECAM-1 level in the OG or adverse pregnancy outcome group showed downregulation in comparison to that in the CG or good pregnancy outcome group. Abnormal endothelial function in HDCP patients can lead to weakened synthesis of PECAM-1 by endothelial cells, while a lack of serum PECAM-1 levels can affect endothelial function and worsen patients’ condition. Herein, PECAM-l decreased as the severity of HDCP deepened. The previous report depicted that PECAM-1 in HDCP patients can participate in the occurrence and development of HDCP, and it changes with the severity of condition (18), which is consistent with the results of this research.

PLGF, expressed in the uterus and placenta, exerts a vital role in endothelial cell proliferation, apoptosis, and elevated vascular permeability (19). Herein, the serum PLGF level in OG was downregulated in comparison to that in CG. PLGF exerts a certain antagonistic influence and is a major cytokine responsible for placental function and vascular formation. Herein, serum PLGF level in the adverse pregnancy outcome group illustrated downregulation in comparison to that in the good pregnancy outcome group, and its expression level tended to decrease as patients’ condition worsened. The previous report has demonstrated that the serum PLGF level in the good pregnancy outcome group depicted a remarkable elevation in comparison to that in the adverse pregnancy outcome group (20), which is consistent with the results of this research.

Research has illustrated that physiological metabolism during pregnancy can lead to deficiencies in different trace elements (iron, zinc, copper, selenium, etc.), thereby affecting changes in gut microbiota (21). The corresponding changes in gut microbiota further lead to microbiota functional disorders during pregnancy, causing the occurrence of related diseases such as HDCP and eclampsia (22). Based on these clear pathogenic mechanisms, serum trace element indicators can be applied as early diagnostic indicators in clinical practice, while monitoring characteristic microbiota populations can be applied as evaluation indicators for efficacy and prognosis. Bifidobacterium and Lactobacillus can facilitate the formation of a healthy intestinal microenvironment, affecting metabolic processes of the body, including ameliorating lipid metabolism and enhancing insulin sensitivity, which helps prevent and control HDCP (23, 24). Herein, Bifidobacterium and Lactobacillus colony counts in the OG, or adverse pregnancy outcome group, illustrated downregulation in comparison to those in the CG, or good pregnancy outcome group, and colony counts tended to decrease as patients’ condition worsened. Our research demonstrates the enormous potential of microbiology in predicting, preventing, and treating pregnancy-related complications.

Through plotting the ROC curves, this research depicted that the AUC of the combined diagnosis of RI, PI, S/D, serum PECAM-1, and PLGF levels, Lactobacillus, Enterococcus, Bifidobacterium, and *E. coli* for predicting adverse pregnancy outcomes in HDCP was 0.878, which illustrated upregulation in comparison to that of individual diagnosis. This suggests that the combined detection of these indicators helps enhance the predictive value of adverse pregnancy outcomes in HDCP pregnant women.

### Limitations

Despite our efforts, this study has several limitations. First, it is a single-center study with a relatively modest sample size, which may limit the generalizability of our findings. Multi-center studies with larger cohorts are warranted validation. Second, while we updated many references to reflect recent advances, the inclusion of some seminal older studies establish foundational concepts (e.g., the initial association of PECAM-1 with pregnancy-induced hypertension) means that the introductory background may not be entirely comprised of the most contemporary literature. However, these classic studies remain critically important for contextualizing our research. Future reviews on this topic would benefit from incorporating an even broader range of the latest mechanistic and clinical studies. Third, observational designs can demonstrate association but not causation. Intervention studies targeting the gut microbiota or PECAM-1/PLGF pathways are needed to establish causal relationships and therapeutic potential.

### Clinical translation and future perspectives

The promising AUC of our combined model underscores its potential as a robust prognostic tool. However, translating this multimodal approach into routine obstetric care necessitates consideration of practical feasibility. Uterine artery Doppler ultrasonography is already a well-established technique in many prenatal care settings. Serum PECAM-1 and PLGF assays are becoming increasingly available in clinical laboratories. The principal challenge lies in the integration of gut microbiota profiling. Current gold-standard methods for precise microbial quantification (e.g., metagenomic sequencing) are time-consuming, costly, and require specialized bioinformatics expertise, which may limit their immediate widespread adoption in all clinical environments. Future research should focus on developing standardized, rapid, and cost-effective assays (e.g., targeted qPCR for key bacterial groups or metabolomic profiling of stool samples) that could more readily be implemented in a clinical workflow. Furthermore, external validation of our model in diverse, multi-ethnic populations is essential before clinical application. Despite these hurdles, our study provides proof-of-concept that integrating vascular, biochemical, and microbial data can significantly enhance risk prediction, paving the way for more personalized monitoring and timely intervention strategies for pregnant women with HDCP.

## Conclusion

A combination of uterine artery Doppler ultrasonography, serum PECAM-1 and PLGF levels, and gut microbiota colony count has high prognostic value for predicting adverse pregnancy outcomes in HDCP, which can provide clinical treatment options and is of great significance for improving pregnancy outcomes in pregnant women. These findings also provide a more informed clinical decision practical tool for nursing assessment, enabling -making and individualized patient management in HDCP care.

## Data Availability

The datasets presented in this study can be found in online repositories. The names of the repository/repositories and accession number(s) can be found in the article/supplementary material.
